# Targeted Alpha Therapy: All We Need to Know about ^225^Ac’s Physical Characteristics and Production as a Potential Theranostic Radionuclide

**DOI:** 10.3390/ph16121679

**Published:** 2023-12-02

**Authors:** Wael Jalloul, Vlad Ghizdovat, Cati Raluca Stolniceanu, Teodor Ionescu, Irena Cristina Grierosu, Ioana Pavaleanu, Mihaela Moscalu, Cipriana Stefanescu

**Affiliations:** 1 Department of Biophysics and Medical Physics-Nuclear Medicine, “Grigore T. Popa” University of Medicine and Pharmacy, 700115 Iasi, Romania; 2 North East Regional Innovative Cluster for Structural and Molecular Imaging (Imago-Mol), 700115 Iasi, Romania; 3 Department of Morpho-Functional Sciences (Pathophysiology), “Grigore T. Popa” University of Medicine and Pharmacy, 700115 Iasi, Romania; 4 Department of Mother and Child, “Grigore T. Popa” University of Medicine and Pharmacy, 700115 Iasi, Romania; 5 Department of Preventive Medicine and Interdisciplinarity, “Grigore T. Popa” University of Medicine and Pharmacy, 700115 Iasi, Romania

**Keywords:** targeted alpha therapy, ^225^Ac, physical properties, production routes, theranostic application

## Abstract

The high energy of α emitters, and the strong linear energy transfer that goes along with it, lead to very efficient cell killing through DNA damage. Moreover, the degree of oxygenation and the cell cycle state have no impact on these effects. Therefore, α radioisotopes can offer a treatment choice to individuals who are not responding to β− or gamma-radiation therapy or chemotherapy drugs. Only a few α-particle emitters are suitable for targeted alpha therapy (TAT) and clinical applications. The majority of available clinical research involves ^225^Ac and its daughter nuclide ^213^Bi. Additionally, the ^225^Ac disintegration cascade generates γ decays that can be used in single-photon emission computed tomography (SPECT) imaging, expanding the potential theranostic applications in nuclear medicine. Despite the growing interest in applying ^225^Ac, the restricted global accessibility of this radioisotope makes it difficult to conduct extensive clinical trials for many radiopharmaceutical candidates. To boost the availability of ^225^Ac, along with its clinical and potential theranostic applications, this review attempts to highlight the fundamental physical properties of this α-particle-emitting isotope, as well as its existing and possible production methods.

## 1. Introduction

At the end of the 1800s, Pierre and Marie Curie, along with Alexander Graham Bell in the early 1900s, conducted research linked to cancer-targeted α therapy (TAT), which represented one of the earliest non-surgical cancer treatments [1]. Furthermore, α-particle emitters have significant curative effects, particularly in patients with limited therapeutic options and metastatic spread [2,3,4]. They can target very small clusters of metastatic cancer cells.

There are many benefits of using these radioisotopes in cancer therapy over common methods. α particles can selectively destroy tumour cells while preserving adjacent normal tissues due to their narrow extent in human tissue, corresponding to less than 0.1 mm [5]. Meanwhile, highly efficient cell destruction through DNA double-strand and DNA cluster damage is caused by the high energy of α emitters, in addition to the strong linear energy transfer (LET) (80 keV/µm) that goes along with it. These effects are mainly unaffected by the state of the cell cycle and oxygenation [6,7,8]. Thus, α radioisotopes can provide a therapeutic option for patients who are resistant to therapy with β− or gamma radiation or chemotherapeutic medications [9,10,11]. According to research estimations, tens of thousands of β− particles are needed to reach a single-cell killing rate of 99.99%, whereas only a few α decays are needed to accomplish a similar killing potential [4,12].

**Figure 1:**
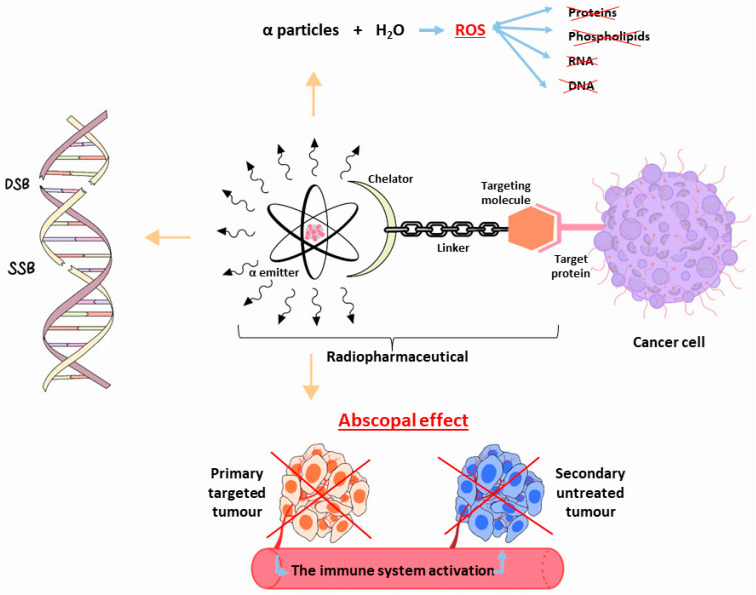
Schematic representation of the biological effects following the use of α-particle emitter radiopharmaceutical for cancer therapy. SSD = Single-Strand Break, DSB = Double-Strand Break, ROS = Reactive Oxygen Species.

The high-LET radiation’s biological efficacy is explained by its tendency to cause complex multiple clusters and double-strand or single-strand breaks in a target cells’ DNA, rendering cellular repair mechanisms ineffective [4,13]. Additionally, reactive oxygen species (ROS), which are produced when emitted particles interact with water, can react with biomolecules such as proteins, phospholipids, RNA, and DNA, leading to permanent cell deterioration [14]. Moreover, during this type of therapy, the primary tumour and any additional cancerous lesions in the body that the radiation did not directly target may decrease as a result of “the abscopal effect” [14]. It is thought that the immune system is a key player in this process, even though the precise biological mechanisms underlying the phenomenon are as yet unknown [4,15,16] (Figure 1).

Considering the clinical application of TAT, only a limited number of α-particle emitters are appropriate [17]. The use of ^225^Ac and its short-lived daughter nuclide ^213^Bi represents the vast majority of available experience in clinical research [5]. Furthermore, applying γ decays, which are produced during the radioactive ^225^Ac cascade [5] in SPECT imaging, raises the possibility of theranostic nuclear medicine applications.

Although interest in using ^225^Ac as an α-emitting radiolabel has been steadily increasing [18], substantial clinical investigations of many radiopharmaceutical candidates cannot be supported due to ^225^Ac’s limited worldwide accessibility [19]. Notwithstanding the significant financial investments made by numerous laboratories to establish production pathways, the widespread use of ^225^Ac-labeled radiopharmaceuticals in human patients is still not achievable [19]. This ongoing shortage in ^225^Ac supply can be explained by the practical production techniques that need difficult logistical tasks, such as using controlled nuclear materials or highly irradiating radioactive accelerator targets [19].

In order to increase the availability of ^225^Ac and thus boosting the clinical use of α-particle-emitter therapeutics and potential theranostic applications, this review aims to outline the fundamental physical characteristics of ^225^Ac in addition to its existing and potential production routes.

## 2. ^225^Ac: Physical Characteristics

Actinium is a radioactive component with atomic number 89 [20]. Only two of its 32 isotopes, ^228^Ac and ^227^Ac, are naturally produced as a result of the disintegration of ^232^Th and ^235^U, respectively [20,21]. With its long half-life of 21.7 years and predominant β− emissions decay, ^227^Ac represents the most common actinium isotope. However, ^228^Ac, which is also a β− emitter, is highly uncommon [20,21].

^225^Ac is the initial element in the actinide family, and its radioactive parents are parts of the now-extinct “neptunium series” [19,21]. This α-emitter isotope has a long half-life of 9.9 days [5,22].

Starting from ^225^Ac to reach ^209^Bi (T_1/2_ = 1.9 × 10^19^ y), the decay series includes six short-lived radionuclide daughters [5,23].

**Figure 2:**
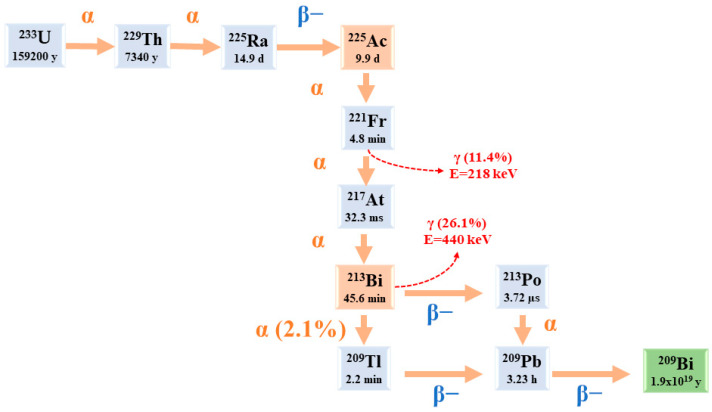
The decay chain of ^233^U to ^225^Ac and ^213^Bi.

This radioactive cascade is represented by ^221^Fr (T_1/2_ = 4.8 min; 6.3 MeV α particle and 218 keV γ emission), ^217^At (T_1/2_ = 32.3 ms; 7.1 MeV α particle), ^213^Bi (T_1/2_ = 45.6 min; 5.9 MeV α particle, 492 keV β− particle and 440 keV γ emission), ^213^Po (T_1/2_ = 3.72 µs; 8.4 MeV α particle), ^209^Tl (T_1/2_ = 2.2 min; 178 keV β− particle), ^209^Pb (T_1/2_ = 3.23 h; 198 keV β− particle) [24] (Figure 2) [14].

## 3. ^225^Ac and Its Potential Theranostic Use

^225^Ac is considered a “nanogenerator”, since one decay of this element produces a total of four α and three β particles, in addition to two γ emissions [24]. Taking into account its α particle emissions, along with the fact that the non-tumour binding activity can be eliminated before most of its dose is deposited in organs, ^225^Ac is considered an appealing choice for TAT [24,25]. However, it is important to give attention to the notable ^225^Ac cytotoxicity, including renal toxicity [26], due to its extended half-life and the various α particles produced throughout its decay chain [5].

A theranostic-based approach, characterised by the imaging–therapeutic duality, is the process of obtaining positron emission tomography (PET) and SPECT scans by exchanging the therapeutic α-emitting radionuclide with a positron or gamma diagnostic imaging radionuclide. Significant information on dosimetry and TAT reactions is obtained from these relevant nuclear medicine images.

Chemical characteristics, half-life, radioactive emission type and intensity, related dosimetry, ease and scalability of production, radionuclidic purity, economics, and radionuclide progeny considerations are the factors that determine “the ideal” imaging surrogates for targeted alpha therapy [27,28].

Therapeutic use of ^225^Ac is often paired with imperfect PET imaging surrogates, such as ^68^Ga, ^89^Zr, or ^111^In, despite significant differences in their half-lives or chelation chemistry [29]. Studies are being conducted to address the limitations of imaging radionuclides by utilising lanthanum (La) as a potential alternative, especially ^132^La (T_1/2_ = 4.8 h, 42% β+) and ^133^La (T_1/2_ = 3.9 h, 7% β+) [30,31]. However, the half-lives of these isotopes are much shorter than that of ^225^Ac, limiting their applicability in PET imaging [29]. In this regard, the production of ^134^Ce (T_1/2_ = 3.2 d) has recently been started by the U.S. Department of Energy (DOE) Isotope Program [32]. The long ^134^Ce T_1/2_ and the similar chemical properties of ^225^Ac and ^134^Ce were considered potential benefits for monitoring in vivo pharmacokinetics. For PET imaging of the chelate and the antibody trastuzumab, ^134^Ce has been demonstrated to bind with diethylenetriamine pentaacetate (DTPA) [32] and dodecane tetraacetic acid (DOTA) [33]. On the other hand, greater molar ratios and higher temperatures are needed for isotope combinations with DOTA and DTPA [29]. In contrast, N, N′-bis[(6-carboxy-2-pyridyl)methyl]-4,13-diaza-18-crown-6 (macropa) has shown great stability for nonradioactive cerium and better chelate characteristics for ^225^Ac [34], indicating that it might be useful for the theranostic development of ^134^Ce/^225^Ac [35].

The potential use of γ disintegrations, obtained by the decay of the intermediate ^221^Fr (218 keV, 11.6% emission probability) and ^213^Bi (440 keV, 26.1% emission probability) [5], in SPECT in vivo imaging could lead the ^225^Ac radioactive cascade to a possible theranostic prospective in nuclear medicine applications. Nonetheless, planar SPECT imaging would be challenging because of the effectiveness of ^225^Ac, which results in modest administered doses (~50–200 kBq/kg [5]), along with low γ emissions [24,25]. As a possible solution to this limitation, we can notice the suitable use of ^213^Bi, which can be isolated from the ^225^Ac decay cascades [24]. Nevertheless, it is mandatory to consider the short half-life of ^213^Bi (45.6 min), which poses difficulties for processing, radiolabelling, and radiopharmaceutical delivery [24]. In addition, it is necessary to point out that these radiations make reaction monitoring complicated. Moreover, the secular equilibrium must be attained (for at least 6 h) before measuring a trustworthy radiochemical yield (RCY) [21]. Actinium’s chemistry lacks advancement because of its restricted availability; all Ac isotopes need specific management and facilities [20].

## 4. Radiochemistry

During the production of radionuclides, it is mandatory to take into consideration a set of important aspects, such as safety, the co-generation of a few long-lived radionuclidic impurities, and adjustability, to enable delivery through clinical sites [27]. Once the target material has been irradiated, potent chemical purification methods are required to isolate the radioisotope [27,36,37,38]. Furthermore, the alpha particle may radiolytically damage the radiopharmaceutical itself, reducing in vivo targeting and producing more radioactive deposits in nontarget tissue. [27].

Since radiopharmaceuticals are considered typical pharmaceuticals, special manuals have been developed in the *European Pharmacopoeia* to deal with quality control issues [39]. Additionally, optimised protocols for preparing ^225^Ac agents in therapeutic doses have been established [40] (Table 1).

## 5. ^225^Ac Radiopharmaceuticals and Clinical Applications

The delivery of the radiopharmaceutical via the circulatory system enables the targeting of both the main tumour and its metastases. Whether a radiopharmaceutical is intended for therapeutic or diagnostic purposes depends on the decay properties of the linked radioisotope. For the purpose of curing, controlling, or palliating symptoms, TAT aims to provide an adequate amount of ionising radiation to intended malignities areas [27]. This means that any TAT agent must have a thorough understanding of its stability, pharmacokinetics, and dosimetry.

Investigations on ^225^Ac have shown potential in treating neuroendocrine tumours, acute myeloid leukaemia, and metastatic prostate cancer, and more radiopharmaceuticals are being developed for other cancer types [46,47,48,49,50,51,52] (Table 2).

The use of ^225^Ac in clinical practice is limited by its low availability. Breaking through this barrier would allow ^225^Ac therapy to spread widely. Automated synthesis and consistent patient doses are essential, regardless of the production route chosen for this α-isotope acquisition. ^225^Ac can be adapted for the commonly accessible DOTA-conjugated peptides for therapy [41], which are already capable of labelling ^177^Lu or ^90^Y. Marc Pretze et al. [71] studied the effectiveness and consistency of the radiosynthesis process for creating ^225^Ac-labelled DOTA-conjugated peptides. Additionally, the research aimed to establish whether this process could be adapted for clinical production purposes through an automated synthesis platform (cassette-based module—Modular-Lab EAZY, Eckert & Ziegler) [72]. After comparing two purification methods, the researchers obtained ^225^Ac-labelled peptides in an RCY of 80–90% for tumour therapy in patients [71]. Thus, the whole process was meticulously validated in accordance with the regulations of the German Pharmaceuticals Act §13.2b, knowing that the estimated costs for the automated synthesis of 1 MBq ^225^Ac is around EUR 300–390, taking into account that the peptides would cost EUR 600–1000, the cassettes would cost EUR 180–200, and the ML EAZY would cost EUR ~30,000 [71].

## 6. The Production Routes of ^225^Ac

As already mentioned, ^225^Ac is part of the ^237^Np disintegration family that has vanished in nature. This radioactive element could be artificially reproduced [21]. In addition to direct production paths, ^225^Ac is conveniently reachable at numerous points along the decay chain, in particular via ^233^U (T_1/2_ =159200 y, 100% α), ^229^Th (T_1/2_ = 7340 y, 100% α), and ^225^Ra (T_1/2_ = 14.9 d, 100% β−) [19]. ^225^Ac possesses many fewer nucleons than other actinide nuclei that are more stable to be employed as production targets, such as ^232^Th and ^226^Ra [19]. Thus, production methods should, with rare exceptions, rely on radioactive decay or greater energy bombardments.

**Figure 3:**
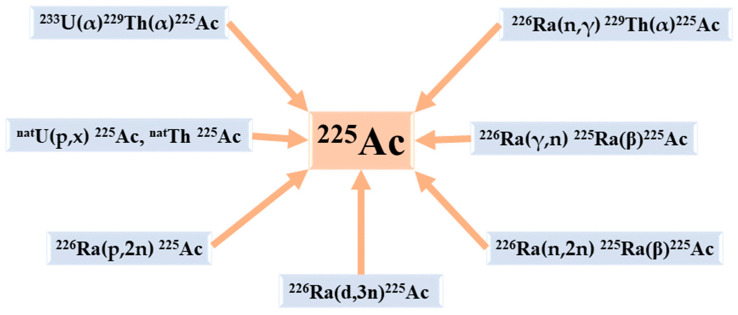
The principal production routes for ^225^Ac.

The available production routes of ^225^Ac and its parents are listed below (Figure 3) [14]:

### 6.1. Radiochemical Extraction from ^229^Th

For more than two decades, the main source of ^225^Ac has been the accumulation of ^229^Th (T_1/2_ = 7340 y) from the disintegration of ^233^U (T_1/2_ = 160,000 y) reserves. At this time, all clinical trials and a large number of pre-clinical studies involving ^225^Ac and ^213^Bi have so far used this type of generation route [5].

A large portion of ^233^U was created between 1954 and 1970 by neutron irradiating ^232^Th when it was being researched for use in nuclear weapons and reactors that were never completely implemented [73,74]. A significant stockpile of ^233^U was kept after the thorium fuel cycle was abandoned in favour of fast reactors powered by plutonium at the end of the 1970s [21]. From supplies kept at the Oak Ridge National Laboratory (ORNL, Oak Ridge, TN, USA), ^229^Th produced via ^233^U disintegration was recovered between 1995 and 2005 [74]. Currently, there are three principal sources for this ^229^Th: at ORNL (5.55 GBq (150 mCi), or 704 mg) [74,75], at the Directorate for Nuclear Safety and Security of the Joint Research Centre (JRC) of the European Commission (JRC, Karlsruhe, Germany) (1.7 GBq (46 mCi), or 215 mg), formerly known as the Institute for Transuranium Elements (ITU) [74,76], and at the Leipunskii Institute for Physics and Power Engineering (IPPE, Obninsk, Russia) (5.55 GBq (150 mCi), or 704 mg) [74,77]. Canadian Nuclear Laboratories (CNL) has more recently announced the isolation of an important ^229^Th source [5]. Very pure sources of ^229^Th were also discovered, prepared, and used for pre-clinical research at the Belgian Nuclear Research Centre (SCK CEN) in Mol, Belgium [14].

By producing approximately 33 GBq (893,23 mCi) (ORNL) [78] and 13.1 GBq (350 mCi) (JRC) [74,76] of ^225^Ac annually, the ORNL and JRC represent, up to now, the principal worldwide providers of ^225^Ac and its parent ^225^Ra (T_1/2_ = 14.9 d). Anion exchange and extraction chromatography are combined to produce ^225^Ac from ^229^Th at JRC Karlsruhe, whereas anion [52] and cation exchange are used in the process at ORNL [78]. Even though the IPPE source has the same amount of ^229^Th as the ORNL source, the recorded values show that the IPPE source intermittently produces ^225^Ac [74,77,79]. According to Samsonov MD et al., IPPE ^225^Ac production could reach 22 GBq per year [80].

Additionally, it has been noted that starting from 2019, a very considerable rise in the availability of ^229^Th will be produced through the extraction of ^229^Th from historical wastes kept by the US DOE [4,52,78]. According to estimations, there could be up to 45 g of total ^229^Th available, which could result in a 40-fold boost in the supply of ^225^Ac above current levels [78].

The ^225^Ac developed at JRC Karlsruhe and ORNL is considered safe for human use and has been significantly utilised for patient treatment [5], although there have been no reports to date about the direct clinical application of ^225^Ac made at IPPE [5].

Approximately 68 GBq of ^225^Ac from ^229^Th are generated per year on a global scale [5]. Knowing that the ^225^Ac-labelled ligands’ given activities typically range from 4 to 50 MBq per therapeutic dosage [5], the amount of this isotope’s supply is sufficient to treat several hundred patients annually and permits the performance of pre-clinical research. Although a major benefit of this production method is that the resulting ^225^Ac is free of other actinium isotopes, the globally generated ^229^Th is not enough to satisfy the extensive use and implementation in healthcare applications across the world [74,81]. Therefore, the development of ^225^Ac radiopharmaceuticals is hindered by the limited supply and high cost that make ^225^Ac inaccessible to many researchers [74]. In addition, the production of ^233^U (T_1/2_ = 160,000 y) is not viewed as a realistic solution for addressing expected short-term ^225^Ac demand, because decades of steady growth are necessary to boost ^229^Th (T_1/2_ = 7340 y) supply [19,82,83]. As a result, numerous other techniques for generating ^225^Ac on a wide scale have been researched.

Exposing radium targets to high fluxes of thermal neutrons is considered an effective procedure to induce ^229^Th production [19]. This approach has been carefully investigated by ORNL researchers with access to the High Flux Isotope Reactor’s (HFIR) > 1015 n cm^−2^ s thermal fluxes, noticing the production of ^229^Th from ^226^Ra, ^228^Ra, and ^227^Ac [19]. An HFIR cycle of 26 days generated ^229^Th yields at 74 ± 7.4 MBq g^−1^ from ^226^Ra, 260 ± 10 Bq g^−1 229^Th from ^228^Ra, and 1200 ± 50 MBq g^−1^ from ^227^Ac [19,84].

^226^Ra(n,γ)^227^Ra(β−)^227^Ac(n,γ)^228^Ac(β−)^228^Th(n,γ)^229^Th is the predominant generation pathway from ^226^Ra targets and is driven by a combination of neutron capture probability and decay kinetics [19]. The short half-lives of ^227^Ra (T_1/2_ = 42.2 min, 100% β−) and ^228^Ac (T_1/2_ = 6.15 h, 100% β−) represent the important restrictions for these possible ^229^Th generation routes [19]. The magnitude of the ^226^Ra(n, γ) ^229^Th cross section has the biggest impact on the amount of ^229^Th that can be produced [19]. Unfortunately, this predominant pathway passes through ^228^Th. This Th isotope is a dosimetrically undesirable contaminant that can only be eliminated from ^229^Th by mass isolation or burnup and lowers the yield of ^229^Th that may be produced [19]. The handling of the radium target and the generation of ^228^Th (T_1/2_ = 1.9 y) intermediate represent important challenges of this process [14,52,85]. In addition, there is still a sizable gap between the theoretically predicted yields and the measured ones. In HFIR, ideal 5-cycle activations are expected to provide approximately 0.8 GBq (20 mCi g^−1^) of ^229^Th for every gram of ^226^Ra [19].

Whereas pure ^227^Ac or ^228^Ra targets are projected to generate somewhat more ^229^Th, the current supply of these radionuclides is substantially less than that of ^226^Ra [19]. Although improving the cost effectiveness of centralised recovery and distribution from ^229^Th stocks, the dedication of even relatively small quantities of ^226^Ra to such irradiations will significantly help to ease the current ^225^Ac shortages. Yet, the full scope of the predicted need cannot be promptly met using this production technique. Thus, other production methods will undoubtedly be pursued simultaneously.

### 6.2. Accelerator-Based Routes

#### 6.2.1. The Spallation of ^232^Th

This method is based on the spallation of ^232^Th to produce ^225^Ac. As a target material, ^232^Th (4.1103 Bq/g, 110 nCi/g) is widely accessible, not excessively radioactive, and presents fewer radiation risks [74]. Many countries are known to have stocks of tens of kilograms of thorium metal and hundreds of tonnes of thorium oxide or thorium nitrate, which are created every year as a byproduct of rare-earth mining and used to make more thorium metal in large amounts [74,86].

Waste recycling of the irradiated ^232^Th target material might not be necessary because of its important accessibility [74].

The irradiation of ^232^Th with highly energetic protons (0.6–2 GeV) accessible at large accelerators has been shown to produce considerable amounts of ^225^Ac [5,87,88]. Production yields of several GBq have been recorded for 10-day irradiations utilising highly energetic proton beams [5,89,90]. From the irradiations of 5 g cm^−2^ targets throughout their roughly 8-month annual running durations, Los Alamos National Laboratory (LANL) can create between 40 and 80 GBq (1–2 Ci) every 10 days. This method is considered to be the most developed production procedure [78] and was validated at the Institute for Nuclear Research (INR), Russian Academy of Sciences (RAS) in Troitsk, Russia, and LANL in the US [78]. Furthermore, the routine use of this technique was introduced by the US DOE Tri-Lab (ORNL, Brookhaven National Laboratory (BNL), LANL) [78]. Once the targets are being handled and the completed product is delivered from ORNL, irradiations can be carried out at BNL (200 MeV at 165 mA) and LANL (100 MeV at 275 mA) [78,91].

The co-production of long-lived ^227^Ac (T_1/2_ = 21.8 y) is the process’ primary constraint [27,78]. A large amount of these radionuclidic impurities is simultaneously produced by the spallation of ^232^Th and needs to be eliminated using the proper multi-step chemical separation methods [5,92,93,94]. The effects of the isotopic impurity on the therapeutic application of the produced ^225^Ac need to be taken into account because ^225^Ac and ^227^Ac cannot be totally chemically separated (0.1–0.2% of the relative activity of ^225^Ac) [21,88]. Even with this limitation, the ^225^Ac produced from high-energy accelerators may still be perfectly suitable for the manufacturing of ^225^Ac/^213^Bi generators, as all actinium daughters will be kept on the generator [14]. According to preliminary research, the ^227^Ac impurity will not significantly affect patient dosimetry [78]. Recently, new purifying techniques that enable a reduction in the ^227^Ac level and the recovery of ^225^Ac with better purity, such as isotope separation (isotope separation on-line (ISOL) at Canada’s particle accelerator centre (TRIUMF)) or a manufacturing method using ^225^Ra produced after the proton irradiation of ^232^Th, have been developed [4,21,95,96,97]. Nonetheless, there are still challenges to be resolved regarding long-lived ^227^Ac licensing and accessibility in medical applications. In addition, due to the 21.8-year half-life, waste management is still a serious issue and will necessitate measures with possibly high related costs.

#### 6.2.2. The Irradiations of ^226^Ra

##### The Proton Irradiation of ^226^Ra

Compared with the ^232^Th spallation reaction, the generation of ^225^Ac from ^226^Ra targets by proton irradiation in a cyclotron has several benefits. This method is based on the reaction ^226^Ra(p,2n)^225^Ac. In medium-sized cyclotrons, at proton energies below 20 MeV (around 16 MeV), this procedure can be carried out with excellent results and at a reasonable cost [5,78,98]. About 5 GBq ^225^Ac, which is comparable to 500 patient doses of 10 MBq ^225^Ac, should be produced after a 24 h exposure of 50 mg ^226^Ra to the highest excitation function at 15–16 MeV with a current of 100 mA protons [78]. It is noteworthy that research, both fundamental and applied, is believed to have relevance to medical cyclotrons that produce radioisotopes at energies between 15 and 25 MeV [14,99].

Since no other long-lived actinium isotopes, such as ^227^Ac, are created during the chemical purification of the irradiation targets, ^225^Ac with high isotopic purity is obtained. By choosing the right proton energies, it is possible to reduce the co-production of the short-lived ^226^Ac (T_1/2_ = 29 h) and ^224^Ac (T_1/2_ = 2.9 h) impurities produced by the reactions ^226^Ra(p,n)^226^Ac and ^226^Ra(p,3n)^224^Ac [5,78]. Furthermore, during the time needed for target cooling and reprocessing, their activity will continue to decrease to low levels. Handling targets that contain milligram amounts of radioactive ^226^Ra (T_1/2_ = 1600 y) and controlling its highly radiotoxic gaseous decay product ^222^Rn (T_1/2_ = 3.8 d) [5,14,98,100] pose significant challenges in the production, processing, and control procedures [5,78]. In addition, due to the limited availability of the target material, it is necessary to consider its recycling process [20]. Currently, facilities in North and South America, Europe, and Asia are researching how to utilise this production strategy. For instance, work on the investigation and development of ^225^Ac generation using ^226^Ra (stored as radioactive waste) has started at the National Institutes for Quantum Science and Technology (QST), Chiba, Japan [100]. These amounts of ^226^Ra have previously been used as a sealed source for brachytherapy. Even in this resource-constrained country, some ^226^Ra was accessible as a target for proton irradiation thanks to the national waste management program [100].

##### The Deuterons’ Irradiation of ^226^Ra

An improved method for producing ^225^Ac, which involves irradiating ^226^Ra with deuterons through the reaction ^226^Ra(d,3n)^225^Ac, has been proposed [101]. Although experimental measurements of the reaction’s cross sections are still in development, simulations indicate that the process will have a greater production yield than the ^226^Ra(p,2n)^225^Ac reaction and a maximum cross section of 864 mb at 18.5 MeV [78]. It is important to consider the prolonged cooling period by the ^226^Ac decay, since deuteron irradiation might result in an increased co-production of ^226^Ac (T_1/2_ = 29 h) [78]. Moreover, there are only a few accelerators that can produce deuteron beams with enough energy.

##### The photonuclear reaction ^226^Ra(γ,n)^225^Ra

The photonuclear reaction ^226^Ra(γ,n)^225^Ra, followed by the beta decay of ^225^Ra to ^225^Ac is a different method for producing ^225^Ac by irradiating ^226^Ra. It is noticed that the photon energy cutoff for the reaction is 6.4 MeV. However, experimentally established cross-section data are not yet available [78]. Modelling data predict modest reaction yields and high-intensity electron beams from modern accelerators are required for commercially viable production. At JRC Karlsruhe, the process’s fundamentals have been experimentally verified [78]. A zircaloy capsule containing 1 mg of ^226^Ra embedded in 800 mg of a BaCl^2^ matrix underwent 3.5 h of 52 MeV betatron irradiation to generate 0.24 mCi of ^225^Ac [78]. At the INR in Dubna, Russia [102], as well as the Illawarra Cancer Centre (ICC) in Wollongong, Australia [103], the procedure’s viability has also been effectively validated. At a maximum photon energy of 24 MeV, a radiation yield of 550 Bq/(mAh mg ^226^Ra) was recorded [102]. For a more precise estimate of production yields, it is extremely important to quantify the cross-section data in detail in this reaction.

The main challenges in this method are the recycling requirement of the ^226^Ra target and some handling issues with the ^222^Rn daughter [20]. However, large-scale ^225^Ac manufacturing using this procedure is already being implemented at several plants [104]. It was reported that SCK CEN is capable of generating high-grade GMP-grade ^225^Ac and also continually supplying it using a backup system [18,100]. During the creation of GMP-grade ^225^Ac, SCK CEN has been collaborating with the Institute of Radioelements Environment & Lifescience Technology (IRE Elit) and Global Morpho Pharma (GMP) (France) [100]. Starting in 2019, SCK-CEN began irradiating their stock of several hundred grammes of ^226^Ra. This Belgian research centre is also equipped with a BR2 reactor and an accelerator-driven subcritical reactor named Multi-purpose hYbrid Research Reactor for High-tech Application (MYRRHA) that are used in this approach [100]. Additionally, utilising an IBA (Ion Beam Applications S.A., EURONEXT) Rhodotron, SCK CEN could produce GMP-grade ^225^Ac at a weekly rate of 37 GBq (1000 mCi) by irradiating with 40 MeV electrons at 125 kW [100]. The prospects should be kept an eye on, as SCK CEN and IBA established a research and development partnership agreement for the joint production of ^225^Ac in 2021 [105] (Table 3).

### 6.3. ^225^Ac/^213^Bi Radionuclide Generators

In the middle of the 1990s, the JRC was the first laboratory to offer ^225^Ac/^213^Bi to clinical partners [5]. Ever since, the JRC has produced these radioisotopes on an annual basis for preclinical research and clinical testing carried out at JRC Karlsruhe or in partnership with a large network of healthcare partners.

In order to produce the short-lived ^213^Bi (T_1/2_ = 45.6 min) on-site, ^225^Ac can either be utilised directly as a therapeutic nuclide [50,106] or set onto ^225^Ac/^213^Bi generators [78,83]. All patient investigations with ^213^Bi up to now have utilised ^225^Ac/^213^Bi generators.

There are numerous generator types available, including those based on ion exchange, extraction chromatography, and inorganic sorbents [106]. The most widely used type is a single-column “direct” generator that was invented at the ITU and based on the strongly acidic cation-exchange sorbent AG MP-50 [106].

**Figure 4:**
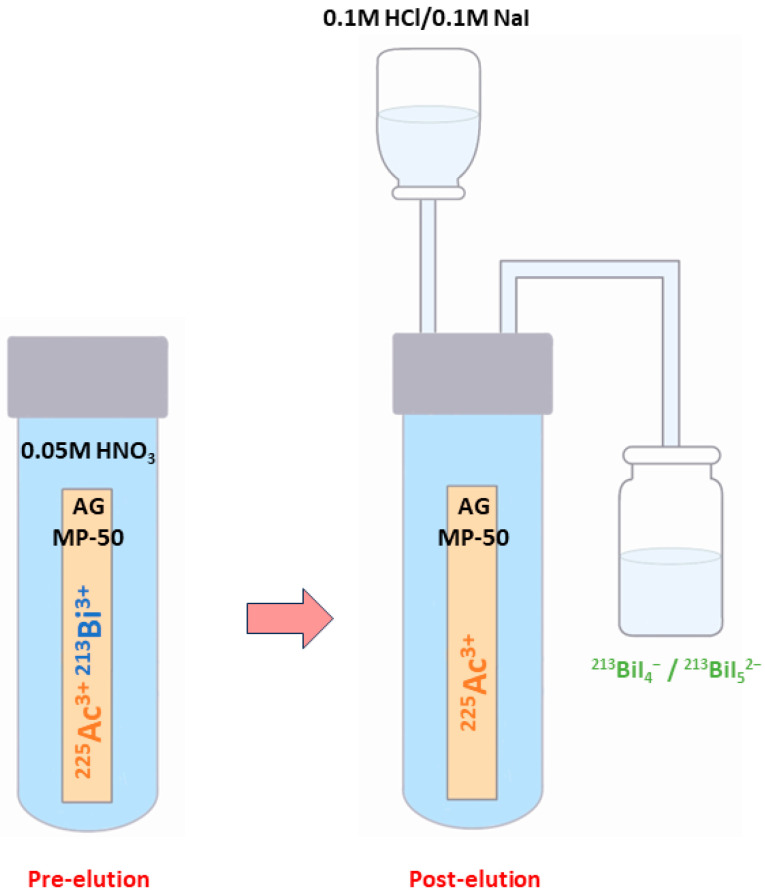
Schematic representation of the ^213^Bi elution using the single-column “direct” ^225^Ac/^213^Bi generator.

In this well-known approach, ^213^Bi is obtained starting from ^225^Ac, which is tightly bound to the sorbent and drowned in 0.05M HNO_3_ solution [14,78,83]. At roughly every 3 h [14,78], ^213^Bi (^213^BiI_4_^-^ and ^213^BiI_5_^2-^) is obtained for immediate use through elution with a mixture of 0.1 M HCl/0.1 M NaI [104] (Figure 4) [14].

The high-activity generator technology created at JRC Karlsruhe enables the generator to function reliably even when supplied with up to 4 GBq ^225^Ac of activities [5,78]. Although the penetration of ^225^Ac is less than 0.2 ppm, the yields of ^213^Bi elution may be more than 80% [107]. The process of distributing ^225^Ac activity uniformly over about two-thirds of the generator resin ensures stable performance over several weeks and minimises radiolytic degradation of the organic resin [5,78].

Injection-ready therapeutic dosages of ^213^Bi-labeled peptides with activities of up to 2.3 GBq have been successfully prepared using the generator for clinical applications [78] including the locoregional therapy of brain tumours [5,13]. Due to the relatively long parent half-life, which enables the transport of the generator to radiopharmacy facilities over vast distances, these generators may be employed clinically.

## 7. Conclusions

Taking into account its α-particle emissions, along with the ability to eliminate the non-tumour binding activity before most of its dose is deposited in organs, ^225^Ac is considered an appealing choice for TAT. Nevertheless, because of its long half-life and the different α particles created throughout its decay chain, it is crucial to pay attention to the considerable cytotoxicity of ^225^Ac. Additionally, the γ disintegrations that result from the intermediate ^221^Fr and ^213^Bi disintegration may be used in SPECT clinical imaging. Thus, the radioactive cascade of ^225^Ac could be used in nuclear medicine, especially in theranostic applications. However, the small ^225^Ac doses given lead to low γ emissions, which makes planar SPECT imaging difficult. A potential alternative for this constraint is to make appropriate use of ^213^Bi, which can be isolated from the decay cascades of ^225^Ac. However, the brief half-life of ^213^Bi must be taken into account since it presents challenges for radiopharmaceutical distribution, processing, and radiolabelling.

Apart from direct production pathways, ^225^Ac can be easily accessed at many points in the decay chain, especially through ^233^U, ^229^Th, and ^225^Ra. Compared with other actinide nuclei, including ^232^Th and ^226^Ra, which are more stable to use as production targets, ^225^Ac has many fewer nucleons. As a result, production techniques must, for the most part, rely on radioactive decay or higher energy bombardments.

All the production techniques discussed in this paper are expensive and will all struggle to satisfy demand at the expected level if they are used separately.

It is necessary to readjust the facilities that are accessible throughout the world, to use suitable production methods that are adapted to the available infrastructure, and take into consideration the advantages and disadvantages of every used production modality. In addition, fruitful collaboration between the different centres and experienced scientific staff will pave the way for the widespread clinical use of actinium-based radiopharmaceuticals as a new standard of care.

The European medical isotope programme: Production of High-Purity Isotopes by Mass Separation Project (PRISMAP) represents an important initiative of this type of collaboration. Coordinated by the European Laboratory for Nuclear Research (CERN), the project partners come from thirteen nations: Austria, Belgium, Denmark, France, Germany, Italy, Latvia, Norway, Portugal, Poland, Sweden, Switzerland, and the United Kingdom. Nine significant EU, national, or regional infrastructures, four developing infrastructures, leader research institutes, medical facilities, the European Joint Research Centre, and one small and midsize enterprise (SME) are among the twenty-three partners that make up the PRISMAP Consortium. With the help of these considerable facilities, the programme goal is to create a sustainable source of high-purity-grade new radionuclides for medical use. It also aims to offer an accessible point of entry for all researchers working in this field, including those from SMEs, global pharmaceutical companies, nuclear centres, hospitals, and universities, by implementing standardised access procedures.

Several PRISMAP partners, including JRC Belgium, Narodowe Centrum Badań Jądrowych (NCBJ), Poland, Institut Max von Laue—Paul Langevin (ILL), France, and SCK CEN, Belgium, are additionally implicated in another promising project in the field of the sustainability of medical isotope production and its safe application in Europe, named the Strengthening the European Chain of sUpply for next-generation medical RadionuclidEs (SECURE). The project focuses on encouraging advancements in the creation of irradiation targets and manufacturing processes for both new and existing isotopes used in nuclear medicine and diagnostics. A list of crucial alpha-emitting radioisotopes in nuclear medicine was created, and ^225^Ac was selected at the top of this list. The research aims to overcome the primary challenges to ensure the future availability of these isotopes by: (1) creating a framework of guidelines and recommendations that enable investigating the full clinical potential of alpha and beta particle therapy and its safe application; (2) offering significant insights that serve as a model for resolving challenges with upscaling and continuous isotope production; (3) removing critical obstacles along the production of specific alpha- and beta-emitting isotopes that restrict a sustainable production.

## Figures and Tables

**Table 1:** Research on ^225^Ac chemistry. RCY = Radiochemical yield, RCP = Radiochemical purity, TLC = Thin-layer chromatography, ITLC = Instant thin-layer chromatography.

Study	Preparation method	Radiopharmaceutical	RCY/RCP
Abou. et al., 2022 [41]	❖ The labelling of the DOTA-conjugated peptide was carried out under good manufacturing practice within a shielded hot cell using a multifunctional automated radiosynthesis module (Trasis, AllinOne mini). ❖ 46.6 MBq of the ^225^Ac source dissolved in 0.2 M HCl was loaded under vacuum in the initial vial for radiolabelling with the DOTA-conjugated precursor (200 µg) on day 5 postsource purification. The source was transferred to the one-pot radiolabelling reactor cassette, in which the reaction occurred in Tris buffer (1 M, pH 7.2) at 85 °C for 70 min in the presence of 20% *v*/*v* L-ascorbic acid at pH 6–8. The radiolabelled peptide was transferred in saline and passed through a 0.2 µm sterilizing filter, resulting in a final volume of 9.7 mL. ❖ The radiolabelled products were characterised using thin-layer chromatography, high-pressure liquid chromatography, gamma counting, and high-energy resolution gamma spectroscopy.	^225^Ac-DOTA-conjugated peptide	>99%/>95%
Dumond. et al., 2022 [42]	❖ PSMA-617 precursor was dissolved in 25 μL metal-free water (0.67 mg/mL) and combined with 500 μL 0.05M Tris buffer, pH 9. ^225^Ac solution (~65 μCi in 15 μL) was added and the reaction was heated at 120 °C for 40–50 min. The resulting reaction was cooled and 0.6 mL gentisic acid solution (4 mg/mL in 0.2 M NH_4_OAc) was added. To formulate the dose for injection, sterile saline (8 mL) was added and the pH was adjusted by the addition of 100 μL 0.05 M Tris buffer (pH 9) to give a final pH of ~7.2. The final solution was filtered using a 0.22 μm GV sterile filter into a sterile dose vial. ❖ Radiochemical purity was determined by radio-TLC (eluent: 50mM sodium citrate, pH 5), and plates were analysed using an AR2000 scanner.	^225^Ac-PSMA-617	>99%/98 ± 1%
Thakral. et al., 2021 [43]	❖ ^225^Ac-PSMA-617 was prepared by adding the peptidic precursor-PSMA-617 (molar ratios, ^225^Ac: PSMA-617 = 30:1) in 1 mL ascorbate buffer to ^225^Ac and heating the reaction mixture at 90 °C for 25 min. ❖ pH was determined using pH paper. ❖ RCP of ^225^Ac-PSMA-617 was determined by ITLC.	^225^Ac-PSMA-617	85–87%/97–99%
Kelly. et al., 2021 [44]	❖ ^225^Ac (9.25 MBq) was obtained from a thorium generator at Canadian Nuclear Laboratories and supplied as the dried [^225^Ac]AcCl_3_ salt. The [^225^Ac]AcCl_3_ was dissolved in 1 mL 1 M NH_4_OAc, pH 7.0, transferred by pipette to a 50 mL centrifuge tube, and diluted to 45 mL in 1 M NH_4_OAc. Stock solution (1 mL), containing approximately 205 kBq [^225^Ac]Ac(OAc)_3_, was transferred by pipette to a plastic Eppendorf tube placed on a digital TermoMixer heating block. Then, 20 µL of the ligand stock solution (0.01–1 mg/mL of PSMA or DOTA or macropa) was added and the reaction was shaken at 300 rpm at either 25 °C or 95 °C. A 3 µL aliquot of the reaction mixture was withdrawn and deposited on the origin of a silica-gel-60-coated aluminium plate (Sigma Aldrich) after incubating the reaction for 1 min, 5 min, and 15 min. ❖ A TLC method was developed to separate the metal complexed ligand from uncomplexed ^225^Ac and its daughter radionuclides.	^225^Ac-PSMA conjugated peptide/ ^225^Ac-DOTA conjugated peptide/ ^225^Ac-macropa conjugated peptide	2.7 ± 0.55%–98.8 ± 0.09%/1.8–99.5%
Hooijman. et al., 2021 [45]	❖ ^225^Ac was diluted into 0.1 M HCl. Stock solutions (10 mL) were proceeded in quartz-coated sterile vials. All purchased chemicals were prepared with Milli-Q water. Stock solutions prepared the day before labelling were 1 M HCl (from 37% HCl), 10 M NaOH, and 0.1 M TRIS-buffer pH 9. Two stock solutions were prepared on the day of labelling: First, 20% ascorbic acid was prepared; the ascorbic acid solution was transformed to ascorbate by the addition of 10 M NaOH to a pH 5.8. Secondly, PSMA-I&T (250 µg) was dissolved in 0.1 M TRIS buffer (pH 9) to a concentration of 600 µg/mL. Directly after labelling, 4 mg/mL diethylenetriaminepentaacetic acid (DTPA) was added to the labelling mixture. A solution for injection was prepared by the addition of ascorbate (50% *v*/*v*) and ethanol (6% *v*/*v*, 96%) into saline.	^225^Ac-PSMA-I&T	>95%/>90%

**Table 2:** Clinical research based on ^225^Ac.

Disease	Study	Radiopharmaceutical
Prostate cancer	Parida et al., 2023 [53]	^225^Ac-PSMA RLT
	Ma et al., 2022 [54]	^225^Ac-PSMA-617
	Sanli et al., 2021 [55]	^225^Ac-PSMA-617
	Sen et al., 2021 [56]	^225^Ac-PSMA-617
	Zacherl et al., 2021 [50]	^225^Ac-PSMA-I&T
	Feuerecker et al., 2021 [57]	^225^Ac-PSMA-617
	Van Der Doelen et al., 2021 [58]	^225^Ac-PSMA-617
	Sathekge et al., 2020 [51]	^225^Ac-PSMA-617
	Yadav et al., 2020 [59]	^225^Ac-PSMA-617
	Satapathy et al., 2020 [60]	^225^Ac-PSMA-617
	Sathekge et al., 2019 [61]	^225^Ac-PSMA-617
	Kratochwil et al., 2018 [62]	^225^Ac-PSMA-617
Neuroendocrine tumours	Ballal et al., 2022 [63]	^225^Ac-DOTATATE
	Yadav et al., 2022 [48]	^225^Ac-DOTATATE
	Kratochwil et al., 2021 [64]	^225^Ac-DOTATATE
	Ballal et al., 2020 [65]	^225^Ac-DOTATATE
	Kratochwil et al., 2015 [66]	^225^Ac-DOTATOC
Acute myeloid leukaemia	Rosenblat et al., 2022 [67]	^225^Ac-lintuzumab
	Jurcic, 2018 [68]	^225^Ac-lintuzumab
	Jurcic et al., 2016 [69]	^225^Ac-lintuzumab
	Jurcic et al., 2011 [70]	^225^Ac-lintuzumab

**Table 3:** Advantages and disadvantages of the potential ^225^Ac production methods.

Production Methods	Advantages	Disadvantages
Radiochemical extraction of ^225^Ac from ^229^Th	❖ A large portion of ^233^U was created by neutron irradiating ^232^Th	❖ ^229^Th and ^233^U have long T_1/2_ values
	❖ A significant stockpile of ^233^U was kept after the thorium fuel cycle was abandoned in favour of fast reactors powered by plutonium	❖ The globally generated ^229^Th is not enough to satisfy the extensive use and implementation in healthcare applications across the world
	❖ The CNL has more recently announced the isolation of an important ^229^Th source	❖ The development of ^225^Ac radiopharmaceuticals is hindered by the limited supply and high cost that make ^225^Ac inaccessible to many researchers
	❖ Very pure sources of ^229^Th were discovered, prepared, and used for pre-clinical research at the SCK CEN	❖ The short half-lives of ^227^Ra and ^228^Ac represent important restrictions for the possible ^226^Ra(n,γ) ^227^Ra(β−) ^227^Ac(n,γ) ^228^Ac(β−) ^228^Th(n,γ) ^229^Th routes
	❖ Starting from 2019, a considerable rise in the availability of ^229^Th will be produced through the extraction of ^229^Th from historical wastes kept by the US DOE	❖ The cross section of ^226^Ra(n, γ) ^229^Th greatly impacts ^229^Th production but is hindered by undesirable contaminant ^228^Th
	❖ The resulting ^225^Ac is free of other actinium isotopes	❖ There is still a sizable gap between the theoretically predicted yields and the measured ones
	❖ Exposing radium targets to high fluxes of thermal neutrons is considered an effective procedure to induce ^229^Th production	❖ Whereas pure ^227^Ac or ^228^Ra targets are projected to generate somewhat more ^229^Th, the current supply of these radionuclides is substantially less than that of ^226^Ra
	❖ Improving the cost effectiveness of centralised recovery and distribution from ^229^Th stocks, the dedication of even relatively small quantities of ^226^Ra to such irradiations will significantly help to ease the current ^225^Ac shortages	❖ The full scope of the predicted need cannot be promptly met using this production technique
Spallation of ^232^Th	❖ ^232^Th is widely accessible and presents fewer radiation risks	❖ The co-production of long-lived ^227^Ac (T_1/2_ = 21.8 y) as a radionuclidic impurity
	❖ Many countries are known to have stocks of tens of kilograms of thorium metal and hundreds of tonnes of thorium oxide or thorium nitrate	❖ ^225^Ac and ^227^Ac cannot be totally chemically separated
	❖ Due to its important accessibility, recycling of ^232^Th target material may not be required	❖ Long-lived ^227^Ac licensing and accessibility in medical applications
	❖ The irradiation of ^232^Th with highly energetic protons has been shown to produce considerable amounts of ^225^Ac	❖ Waste management is still a serious issue and will necessitate measures with possibly high related costs
	❖ It is considered to be the most developed production procedure	
	❖ It is suitable for the manufacturing of ^225^Ac/^213^Bi generators, as all actinium daughters will be kept on the generator	
Proton irradiation of ^226^Ra	❖ In medium-sized cyclotrons, at proton energies below 20 MeV (around 16 MeV), this procedure can be carried out with excellent results and at a reasonable cost	❖ Handling targets that contain milligram amounts of radioactive ^226^Ra (T_1/2_ = 1600 y) and controlling its highly radiotoxic gaseous decay product ^222^Rn (T_1/2_ = 3.8 d)
	❖ About 5 GBq ^225^Ac (500 patient doses of 10 MBq ^225^Ac) should be produced after 24 h exposure of 50 mg ^226^Ra	❖ The limited availability of the target material necessitates its recycling
	❖ Fundamental and applied research is thought to apply to medical cyclotrons that generate radioisotopes	
	❖ No other long-lived actinium isotopes, such as ^227^Ac, are created during the chemical purification of the irradiation targets, thus ^225^Ac with high isotopic purity is obtained	
Deuterons’ irradiation of ^226^Ra	❖ Simulations indicate that the process will have a greater production yield than the ^226^Ra(p,2n)^225^Ac reaction	❖ Experimental measurements of the reaction’s cross sections are still in development
		❖ To consider a prolonged cooling period by the ^226^Ac decay since deuteron irradiation might result in an increased co-production of ^226^Ac
		❖ There are a few accelerators that can produce deuteron beams with enough energy
Photonuclear reaction ^226^Ra(γ,n) ^225^Ra	❖ Large-scale ^225^Ac manufacturing using this procedure is already being implemented at several plants	❖ Experimentally established cross-section data are not yet available
		❖ Modest reaction yields are predicted by modelling data
		❖ For commercially feasible production, modern accelerators with high-intensity electron beams are needed
		❖ The recycling requirement of the ^226^Ra target
		❖ Issues with handling the ^222^Rn daughter

## Data Availability

Not applicable.

## Abbreviations

Targeted Alpha Therapy **(TAT)**, Single-Photon Emission Computed Tomography **(SPECT)**, Linear Energy Transfer **(LET)**, Reactive Oxygen Species **(ROS)**, Single-Strand Break **(SSD)**, Double-Strand Break **(DSB)**, Positron Emission Tomography **(PET)**, Department of Energy **(DOE)**, Diethylenetriamine Pentaacetate **(DTPA)**, Dodecane Tetraacetic Acid **(DOTA)**, N,N′-Bis[(6-carboxy-2-pyridyl)methyl]-4,13-diaza-18-crown-6 **(macropa)**, Radiochemical Yield **(RCY)**, Radiochemical Purity **(RCP)**, Thin-Layer Chromatography **(TLC)**, Instant Thin-Layer Chromatography **(ITLC)**, Oak Ridge National Laboratory **(ORNL)**, Directorate for Nuclear Safety and Security of the Joint Research Centre **(JRC)**, Institute for Transuranium Elements **(ITU)**, Leipunskii Institute for Physics and Power Engineering **(IPPE)**, Canadian Nuclear Laboratories **(CNL)**, Belgian Nuclear Research Centre **(SCK CEN)**, High Flux Isotope Reactor **(HFIR)**, Los Alamos National Laboratory **(LANL)**, Institute for Nuclear Research **(INR)**, Russian Academy of Sciences **(RAS)**, Brookhaven National Laboratory **(BNL)**, Isotope Separation On-Line **(ISOL)**, Canada’s Particle Accelerator Centre **(TRIUMF)**, National Institutes for Quantum Science and Technology **(QST)**, Illawarra Cancer Centre **(ICC)**, Institute of Radioelements Environment & Lifescience Technology **(IRE Elit)**, Global Morpho Pharma **(GMP)**, Multi-purpose hYbrid Research Reactor for High-tech Application **(MYRRHA)**, Ion Beam Applications S.A. **(IBA)**, The European Medical Isotope Programme: Production of High-Purity Isotopes by Mass Separation **(PRISMAP)**, European Laboratory for Nuclear Research **(CERN)**, Small and Midsize Enterprise **(SME)**, Narodowe Centrum Badań Jądrowych **(NCBJ)**, Institut Max von Laue—Paul Langevin **(ILL)**, Strengthening the European Chain of sUpply for next generation medical RadionuclidEs **(SECURE)**.

## References

[1] McDevitt M.R., Sgouros G., Sofou S. (2018). Targeted and Nontargeted α-Particle Therapies. Annu. Rev. Biomed. Eng..

[2] Parker C., Nilsson S., Heinrich D., Helle S.I., O’Sullivan J.M., Fosså S.D., Chodacki A., Wiechno P., Logue J., Seke M. (2013). Alpha Emitter Radium-223 and Survival in Metastatic Prostate Cancer. N. Engl. J. Med..

[3] Jurcic J.G., Ravandi F., Pagel J.M., Park J.H., Smith B.D., Douer D., Estey E.H., Kantarjian H.M., Wahl R.L., Earle D. (2014). Phase I Trial of Targeted Alpha-Particle Therapy Using Actinium-225 (^225^Ac)-Lintuzumab (Anti-CD33) in Combination with Low-Dose Cytarabine (LDAC) for Older Patients with Untreated Acute Myeloid Leukemia (AML). Blood.

[4] Johnson J.D., Heines M., Bruchertseifer F., Chevallay E., Cocolios T.E., Dockx K., Duchemin C., Heinitz S., Heinke R., Hurier S. (2023). Resonant Laser Ionization and Mass Separation of ^225^Ac. Sci. Rep..

[5] Morgenstern A., Apostolidis C., Kratochwil C., Sathekge M., Krolicki L., Bruchertseifer F. (2018). An Overview of Targeted Alpha Therapy with ^225^Actinium and ^213^Bismuth. Curr. Radiopharm..

[6] Sgouros G., Roeske J.C., McDevitt M.R., Palm S., Allen B.J., Fisher D.R., Brill A.B., Song H., Howell R.W., Akabani G. (2010). MIRD Pamphlet No. 22 (Abridged): Radiobiology and Dosimetry of Alpha-Particle Emitters for Targeted Radionuclide Therapy. J. Nucl. Med..

[7] Wulbrand C., Seidl C., Gaertner F.C., Bruchertseifer F., Morgenstern A., Essler M., Senekowitsch-Schmidtke R. (2013). Alpha-Particle Emitting ^213^Bi-Anti-EGFR Immunoconjugates Eradicate Tumor Cells Independent of Oxygenation. PLoS ONE.

[8] Elgqvist J., Frost S., Pouget J.-P., Albertsson P. (2014). The Potential and Hurdles of Targeted Alpha Therapy—Clinical Trials and Beyond. Front. Oncol..

[9] Friesen C., Glatting G., Koop B., Schwarz K., Morgenstern A., Apostolidis C., Debatin K.-M., Reske S.N. (2007). Breaking Chemoresistance and Radioresistance with [^213^Bi]Anti-CD45 Antibodies in Leukemia Cells. Cancer Res..

[10] Kratochwil C., Giesel F.L., Bruchertseifer F., Mier W., Apostolidis C., Boll R., Murphy K., Haberkorn U., Morgenstern A. (2014). ^213^Bi-DOTATOC Receptor-Targeted Alpha-Radionuclide Therapy Induces Remission in Neuroendocrine Tumours Refractory to Beta Radiation: A First-in-Human Experience. Eur. J. Nucl. Med. Mol. Imaging.

[11] Kratochwil C., Bruchertseifer F., Giesel F.L., Weis M., Verburg F.A., Mottaghy F., Kopka K., Apostolidis C., Haberkorn U., Morgenstern A. (2016). ^225^Ac-PSMA-617 for PSMA-Targeted α-Radiation Therapy of Metastatic Castration-Resistant Prostate Cancer. J. Nucl. Med..

[12] Humm J.L., Cobb L.M. (1990). Nonuniformity of Tumor Dose in Radioimmunotherapy. J. Nucl. Med..

[13] Guerra Liberal F.D.C., O’Sullivan J.M., McMahon S.J., Prise K.M. (2020). Targeted Alpha Therapy: Current Clinical Applications. Cancer Biother. Radiopharm..

[14] Ahenkorah S., Cassells I., Deroose C.M., Cardinaels T., Burgoyne A.R., Bormans G., Ooms M., Cleeren F. (2021). Bismuth-213 for Targeted Radionuclide Therapy: From Atom to Bedside. Pharmaceutics.

[15] Vermeulen K., Vandamme M., Bormans G., Cleeren F. (2019). Design and Challenges of Radiopharmaceuticals. Semin. Nucl. Med..

[16] Beyls C., Haustermans K., Deroose C.M., Pans S., Vanbeckevoort D., Verslype C., Dekervel J. (2020). Could Autoimmune Disease Contribute to the Abscopal Effect in Metastatic Hepatocellular Carcinoma?. Hepatology.

[17] Seidl C. (2014). Radioimmunotherapy with α-Particle-Emitting Radionuclides. Immunotherapy.

[18] Zimmermann R. (2023). Is Actinium Really Happening?. J. Nucl. Med..

[19] Engle J.W. (2018). The Production of Ac-225. Curr. Radiopharm..

[20] Hatcher-Lamarre J.L., Sanders V.A., Rahman M., Cutler C.S., Francesconi L.C. (2021). Alpha Emitting Nuclides for Targeted Therapy. Nucl. Med. Biol..

[21] Eychenne R., Chérel M., Haddad F., Guérard F., Gestin J.-F. (2021). Overview of the Most Promising Radionuclides for Targeted Alpha Therapy: The “Hopeful Eight”. Pharmaceutics.

[22] Pommé S., Marouli M., Suliman G., Dikmen H., Van Ammel R., Jobbágy V., Dirican A., Stroh H., Paepen J., Bruchertseifer F. (2012). Measurement of the ^225^Ac Half-Life. Appl. Radiat. Isot..

[23] Suliman G., Pommé S., Marouli M., Van Ammel R., Stroh H., Jobbágy V., Paepen J., Dirican A., Bruchertseifer F., Apostolidis C. (2013). Half-Lives of ^221^Fr, ^217^At, ^213^Bi, ^213^Po and ^209^Pb from the ^225^Ac Decay Series. Appl. Radiat. Isot..

[24] Nelson B.J.B., Andersson J.D., Wuest F. (2020). Targeted Alpha Therapy: Progress in Radionuclide Production, Radiochemistry, and Applications. Pharmaceutics.

[25] Scheinberg D.A., McDevitt M.R. (2011). Actinium-225 in Targeted Alpha-Particle Therapeutic Applications. Curr. Radiopharm..

[26] Muslimov A.R., Antuganov D., Tarakanchikova Y.V., Karpov T.E., Zhukov M.V., Zyuzin M.V., Timin A.S. (2021). An Investigation of Calcium Carbonate Core-Shell Particles for Incorporation of ^225^Ac and Sequester of Daughter Radionuclides: In Vitro and in Vivo Studies. J. Control Release.

[27] Nelson B.J.B., Wilson J., Andersson J.D., Wuest F. (2023). Theranostic Imaging Surrogates for Targeted Alpha Therapy: Progress in Production, Purification, and Applications. Pharmaceuticals.

[28] Saini S., Bartels J.L., Appiah J.-P.K., Rider J.H., Baumhover N., Schultz M.K., Lapi S.E. (2023). Optimized Methods for the Production of High-Purity 203Pb Using Electroplated Thallium Targets. J. Nucl. Med..

[29] Bobba K.N., Bidkar A.P., Meher N., Fong C., Wadhwa A., Dhrona S., Sorlin A., Bidlingmaier S., Shuere B., He J. (2023). Evaluation of ^134^Ce/^13^4La as a PET Imaging Theranostic Pair for ^225^Ac α-Radiotherapeutics. J. Nucl. Med..

[30] Aluicio-Sarduy E., Barnhart T.E., Weichert J., Hernandez R., Engle J.W. (2021). Cyclotron-Produced 132La as a PET Imaging Surrogate for Therapeutic ^225^Ac. J. Nucl. Med..

[31] Nelson B.J.B., Ferguson S., Wuest M., Wilson J., Duke M.J.M., Richter S., Soenke-Jans H., Andersson J.D., Juengling F., Wuest F. (2022). First In Vivo and Phantom Imaging of Cyclotron-Produced ^133^La as a Theranostic Radionuclide for ^225^Ac and ^135^La. J. Nucl. Med..

[32] Bailey T.A., Mocko V., Shield K.M., An D.D., Akin A.C., Birnbaum E.R., Brugh M., Cooley J.C., Engle J.W., Fassbender M.E. (2021). Developing the ^134^Ce and ^134^La Pair as Companion Positron Emission Tomography Diagnostic Isotopes for ^225^Ac and ^227^Th Radiotherapeutics. Nat. Chem..

[33] Bailey T.A., Wacker J.N., An D.D., Carter K.P., Davis R.C., Mocko V., Larrabee J., Shield K.M., Lam M.N., Booth C.H. (2022). Evaluation of ^134^Ce as a PET Imaging Surrogate for Antibody Drug Conjugates Incorporating ^225^Ac. Nucl. Med. Biol..

[34] Hu A., Aluicio-Sarduy E., Brown V., MacMillan S.N., Becker K.V., Barnhart T.E., Radchenko V., Ramogida C.F., Engle J.W., Wilson J.J. (2021). Py-Macrodipa: A Janus Chelator Capable of Binding Medicinally Relevant Rare-Earth Radiometals of Disparate Sizes. J. Am. Chem. Soc..

[35] Thiele N.A., Brown V., Kelly J.M., Amor-Coarasa A., Jermilova U., MacMillan S.N., Nikolopoulou A., Ponnala S., Ramogida C.F., Robertson A.K.H. (2017). An Eighteen-Membered Macrocyclic Ligand for Actinium-225 Targeted Alpha Therapy. Angew. Chem. Int. Ed. Engl..

[36] Rizk H.E., Breky M.M.E., Attallah M.F. (2023). Development of Purification of No-Carrier-Added 47Sc of Theranostic Interest: Selective Separation Study from the natTi(n,p) Process. Radiochim. Acta.

[37] Mousa A.M., Abdel Aziz O.A., Al-Hagar O.E.A., Gizawy M.A., Allan K.F., Attallah M.F. (2020). Biosynthetic New Composite Material Containing CuO Nanoparticles Produced by Aspergillus Terreus for 47Sc Separation of Cancer Theranostics Application from Irradiated Ca Target. Appl. Radiat. Isot..

[38] Attallah M.F., Rizk S.E., Shady S.A. (2018). Separation of 152+154Eu, 90Sr from Radioactive Waste Effluent Using Liquid–Liquid Extraction by Polyglycerol Phthalate. Nucl. Sci. Tech..

[39] Hooijman E.L., Ntihabose C.M., Reuvers T.G.A., Nonnekens J., Aalbersberg E.A., van de Merbel J.R.J.P., Huijmans J.E., Koolen S.L.W., Hendrikx J.J.M.A., de Blois E. (2022). Radiolabeling and Quality Control of Therapeutic Radiopharmaceuticals: Optimization, Clinical Implementation and Comparison of Radio-TLC/HPLC Analysis, Demonstrated by [^177^Lu]Lu-PSMA. EJNMMI Radiopharm. Chem..

[40] Mdanda S., Ngema L.M., Mdlophane A., Sathekge M.M., Zeevaart J.R. (2023). Recent Innovations and Nano-Delivery of Actinium-225: A Narrative Review. Pharmaceutics.

[41] Abou D.S., Zerkel P., Robben J., McLaughlin M., Hazlehurst T., Morse D., Wadas T.J., Pandya D.N., Oyama R., Gaehle G. (2022). Radiopharmaceutical Quality Control Considerations for Accelerator-Produced Actinium Therapies. Cancer Biother. Radiopharm..

[42] Dumond A.R.S., Rodnick M.E., Piert M.R., Scott P.J.H. (2022). Synthesis of ^225^Ac-PSMA-617 for Preclinical Use. Curr. Radiopharm..

[43] Thakral P., Simecek J., Marx S., Kumari J., Pant V., Sen I.B. (2021). In-House Preparation and Quality Control of Ac-225 Prostate-Specific Membrane Antigen-617 for the Targeted Alpha Therapy of Castration-Resistant Prostate Carcinoma. Indian. J. Nucl. Med..

[44] Kelly J.M., Amor-Coarasa A., Sweeney E., Wilson J.J., Causey P.W., Babich J.W. (2021). A Suitable Time Point for Quantifying the Radiochemical Purity of ^225^Ac-Labeled Radiopharmaceuticals. EJNMMI Radiopharm. Chem..

[45] Hooijman E.L., Chalashkan Y., Ling S.W., Kahyargil F.F., Segbers M., Bruchertseifer F., Morgenstern A., Seimbille Y., Koolen S.L.W., Brabander T. (2021). Development of [^225^Ac]Ac-PSMA-I&T for Targeted Alpha Therapy According to GMP Guidelines for Treatment of mCRPC. Pharmaceutics.

[46] Busslinger S.D., Tschan V.J., Richard O.K., Talip Z., Schibli R., Müller C. (2022). [^225^Ac]Ac-SibuDAB for Targeted Alpha Therapy of Prostate Cancer: Preclinical Evaluation and Comparison with [^225^Ac]Ac-PSMA-617. Cancers.

[47] King A.P., Gutsche N.T., Raju N., Fayn S., Baidoo K.E., Bell M.M., Olkowski C.S., Swenson R.E., Lin F.I., Sadowski S.M. (2023). ^225^Ac-MACROPATATE: A Novel α-Particle Peptide Receptor Radionuclide Therapy for Neuroendocrine Tumors. J. Nucl. Med..

[48] Yadav M.P., Ballal S., Sahoo R.K., Bal C. (2022). Efficacy and Safety of ^225^Ac-DOTATATE Targeted Alpha Therapy in Metastatic Paragangliomas: A Pilot Study. Eur. J. Nucl. Med. Mol. Imaging.

[49] Rathke H., Bruchertseifer F., Kratochwil C., Keller H., Giesel F.L., Apostolidis C., Haberkorn U., Morgenstern A. (2021). First Patient Exceeding 5-Year Complete Remission after ^225^Ac-PSMA-TAT. Eur. J. Nucl. Med. Mol. Imaging.

[50] Zacherl M.J., Gildehaus F.J., Mittlmeier L., Böning G., Gosewisch A., Wenter V., Unterrainer M., Schmidt-Hegemann N., Belka C., Kretschmer A. (2021). First Clinical Results for PSMA-Targeted α-Therapy Using ^225^Ac-PSMA-I&T in Advanced-mCRPC Patients. J. Nucl. Med..

[51] Sathekge M., Bruchertseifer F., Vorster M., Lawal I.O., Knoesen O., Mahapane J., Davis C., Reyneke F., Maes A., Kratochwil C. (2020). Predictors of Overall and Disease-Free Survival in Metastatic Castration-Resistant Prostate Cancer Patients Receiving ^225^Ac-PSMA-617 Radioligand Therapy. J. Nucl. Med..

[52] Camacaro J.F., Dunckley C.P., Harman S.E., Fitzgerald H.A., Lakes A.L., Liao Z., Ludwig R.C., McBride K.M., Yalcintas Bethune E., Younes A. (2023). Development of ^225^Ac Production from Low Isotopic Dilution 229Th. ACS Omega.

[53] Parida G.K., Panda R.A., Bishnoi K., Agrawal K. (2023). Efficacy and Safety of Actinium-225 Prostate-Specific Membrane Antigen Radioligand Therapy in Metastatic Prostate Cancer: A Systematic Review and Metanalysis. Med. Princ. Pract..

[54] Ma J., Li L., Liao T., Gong W., Zhang C. (2022). Efficacy and Safety of ^225^Ac-PSMA-617-Targeted Alpha Therapy in Metastatic Castration-Resistant Prostate Cancer: A Systematic Review and Meta-Analysis. Front. Oncol..

[55] Sanli Y., Kuyumcu S., Simsek D.H., Büyükkaya F., Civan C., Isik E.G., Ozkan Z.G., Basaran M., Sanli O. (2021). ^225^Ac-Prostate-Specific Membrane Antigen Therapy for Castration-Resistant Prostate Cancer: A Single-Center Experience. Clin. Nucl. Med..

[56] Sen I., Thakral P., Tiwari P., Pant V., Das S.S., Manda D., Raina V. (2021). Therapeutic Efficacy of ^225^Ac-PSMA-617 Targeted Alpha Therapy in Patients of Metastatic Castrate Resistant Prostate Cancer after Taxane-Based Chemotherapy. Ann. Nucl. Med..

[57] Feuerecker B., Tauber R., Knorr K., Heck M., Beheshti A., Seidl C., Bruchertseifer F., Pickhard A., Gafita A., Kratochwil C. (2021). Activity and Adverse Events of Actinium-225-PSMA-617 in Advanced Metastatic Castration-Resistant Prostate Cancer After Failure of Lutetium-177-PSMA. Eur. Urol..

[58] Van der Doelen M.J., Mehra N., van Oort I.M., Looijen-Salamon M.G., Janssen M.J.R., Custers J.A.E., Slootbeek P.H.J., Kroeze L.I., Bruchertseifer F., Morgenstern A. (2021). Clinical Outcomes and Molecular Profiling of Advanced Metastatic Castration-Resistant Prostate Cancer Patients Treated with ^225^Ac-PSMA-617 Targeted Alpha-Radiation Therapy. Urol. Oncol..

[59] Yadav M.P., Ballal S., Sahoo R.K., Tripathi M., Seth A., Bal C. (2020). Efficacy and Safety of ^225^Ac-PSMA-617 Targeted Alpha Therapy in Metastatic Castration-Resistant Prostate Cancer Patients. Theranostics.

[60] Satapathy S., Mittal B.R., Sood A., Das C.K., Singh S.K., Mavuduru R.S., Bora G.S. (2020). Health-Related Quality-of-Life Outcomes with Actinium-225-Prostate-Specific Membrane Antigen-617 Therapy in Patients with Heavily Pretreated Metastatic Castration-Resistant Prostate Cancer. Indian. J. Nucl. Med..

[61] Sathekge M., Bruchertseifer F., Knoesen O., Reyneke F., Lawal I., Lengana T., Davis C., Mahapane J., Corbett C., Vorster M. (2019). ^225^Ac-PSMA-617 in Chemotherapy-Naive Patients with Advanced Prostate Cancer: A Pilot Study. Eur. J. Nucl. Med. Mol. Imaging.

[62] Kratochwil C., Bruchertseifer F., Rathke H., Hohenfellner M., Giesel F.L., Haberkorn U., Morgenstern A. (2018). Targeted α-Therapy of Metastatic Castration-Resistant Prostate Cancer with ^225^Ac-PSMA-617: Swimmer-Plot Analysis Suggests Efficacy Regarding Duration of Tumor Control. J. Nucl. Med..

[63] Ballal S., Yadav M.P., Tripathi M., Sahoo R.K., Bal C. (2022). Survival Outcomes in Metastatic Gastroenteropancreatic Neuroendocrine Tumor Patients Receiving Concomitant ^225^Ac-DOTATATE Targeted Alpha Therapy and Capecitabine: A Real-World Scenario Management Based Long-Term Outcome Study. J. Nucl. Med..

[64] Kratochwil C., Apostolidis L., Rathke H., Apostolidis C., Bicu F., Bruchertseifer F., Choyke P.L., Haberkorn U., Giesel F.L., Morgenstern A. (2021). Dosing ^225^Ac-DOTATOC in Patients with Somatostatin-Receptor-Positive Solid Tumors: 5-Year Follow-up of Hematological and Renal Toxicity. Eur. J. Nucl. Med. Mol. Imaging.

[65] Ballal S., Yadav M.P., Bal C., Sahoo R.K., Tripathi M. (2020). Broadening Horizons with ^225^Ac-DOTATATE Targeted Alpha Therapy for Gastroenteropancreatic Neuroendocrine Tumour Patients Stable or Refractory to ^177^Lu-DOTATATE PRRT: First Clinical Experience on the Efficacy and Safety. Eur. J. Nucl. Med. Mol. Imaging.

[66] Kratochwil C., Bruchertseifer F., Giesel F., Apostolidis C., Haberkorn U., Morgenstern A. (2015). Ac-225-DOTATOC—An Empiric Dose Finding for Alpha Particle Emitter Based Radionuclide Therapy of Neuroendocrine Tumors. J. Nucl. Med..

[67] Rosenblat T.L., McDevitt M.R., Carrasquillo J.A., Pandit-Taskar N., Frattini M.G., Maslak P.G., Park J.H., Douer D., Cicic D., Larson S.M. (2022). Treatment of Patients with Acute Myeloid Leukemia with the Targeted Alpha-Particle Nanogenerator Actinium-225-Lintuzumab. Clin. Cancer Res..

[68] Jurcic J.G. (2018). Clinical Studies with Bismuth-213 and Actinium-225 for Hematologic Malignancies. Curr. Radiopharm..

[69] Jurcic J.G., Levy M.Y., Park J.H., Ravandi F., Perl A.E., Pagel J.M., Smith B.D., Estey E.H., Kantarjian H., Cicic D. (2016). Phase I Trial of Targeted Alpha-Particle Therapy with Actinium-225 (^225^Ac)-Lintuzumab and Low-Dose Cytarabine (LDAC) in Patients Age 60 or Older with Untreated Acute Myeloid Leukemia (AML). Blood.

[70] Jurcic J.G., Rosenblat T.L., McDevitt M.R., Pandit-Taskar N., Carrasquillo J.A., Chanel S.M., Zikaras K., Frattini M.G., Maslak P.G., Cicic D. (2011). Phase I Trial of the Targeted Alpha-Particle Nano-Generator Actinium-225 (^225^Ac)-Lintuzumab (Anti-CD33; HuM195) in Acute Myeloid Leukemia (AML). Blood.

[71] Pretze M., Kunkel F., Runge R., Freudenberg R., Braune A., Hartmann H., Schwarz U., Brogsitter C., Kotzerke J. (2021). Ac-EAZY! Towards GMP-Compliant Module Syntheses of ^225^Ac-Labeled Peptides for Clinical Application. Pharmaceuticals.

[72] Eryilmaz K., Kilbas B. (2021). Fully-Automated Synthesis of ^177^Lu Labelled FAPI Derivatives on the Module Modular Lab-Eazy. EJNMMI Radiopharm. Chem..

[73] Alvarez R. (2013). Managing the Uranium-233 Stockpile of the United States. Sci. Glob. Secur..

[74] Robertson A.K.H., Ramogida C.F., Schaffer P., Radchenko V. (2018). Development of ^225^Ac Radiopharmaceuticals: TRIUMF Perspectives and Experiences. Curr. Radiopharm..

[75] Boll R.A., Malkemus D., Mirzadeh S. (2005). Production of Actinium-225 for Alpha Particle Mediated Radioimmunotherapy. Appl. Radiat. Isot..

[76] Apostolidis C., Molinet R., Rasmussen G., Morgenstern A. (2005). Production of Ac-225 from Th-229 for Targeted Alpha Therapy. Anal. Chem..

[77] Kotovskii A.A., Nerozin N.A., Prokof’ev I.V., Shapovalov V.V., Yakovshchits Y.A., Bolonkin A.S., Dunin A.V. (2015). Isolation of Actinium-225 for Medical Purposes. Radiochemistry.

[78] Morgenstern A., Apostolidis C., Bruchertseifer F. (2020). Supply and Clinical Application of Actinium-225 and Bismuth-213. Semin. Nucl. Med..

[79] Harvey J.T., Nolen J., Vandergrift G., Gomes I., Kroc T., Horwitz P., McAlister D., Bowers D., Sullivan V., Greene J. (2011). Production of Actinium-225 via High Energy Proton Induced Spallation of Thorium-232.

[80] Samsonov M.D., Nerozin N.A., Podsoblyaev D.A., Prokof’ev I.V., Tkachev S.V., Khamianov S.V., Shapovalov V.V. Isolation of Alpha-Emitting Radionuclides for Nuclear Medicine in JSC “SSC RF–IPPE. Proceedings of the 10th International Symposium on Targeted Alpha Therapy.

[81] USDOE Office of Science (SC) (2015). Meeting Isotope Needs and Capturing Opportunities for the Future: The 2015 Long. Range Plan. for the DOE-NP Isotope Progarm, NSAC Isotopes Subcommitee, July 2015.

[82] Makvandi M., Dupis E., Engle J.W., Nortier F.M., Fassbender M.E., Simon S., Birnbaum E.R., Atcher R.W., John K.D., Rixe O. (2018). Alpha-Emitters and Targeted Alpha Therapy in Oncology: From Basic Science to Clinical Investigations. Target. Oncol..

[83] Morgenstern A., Bruchertseifer F., Apostolidis C. (2012). Bismuth-213 and Actinium-225—Generator Performance and Evolving Therapeutic Applications of Two Generator-Derived Alpha-Emitting Radioisotopes. Curr. Radiopharm..

[84] Hogle S., Boll R.A., Murphy K., Denton D., Owens A., Haverlock T.J., Garland M., Mirzadeh S. (2016). Reactor Production of Thorium-229. Appl. Radiat. Isot..

[85] Kratochwil C., Bruchertseifer F., Rathke H., Bronzel M., Apostolidis C., Weichert W., Haberkorn U., Giesel F.L., Morgenstern A. (2017). Targeted α-Therapy of Metastatic Castration-Resistant Prostate Cancer with ^225^Ac-PSMA-617: Dosimetry Estimate and Empiric Dose Finding. J. Nucl. Med..

[86] Englert M., Krall L., Ewing R.C. (2012). Is Nuclear Fission a Sustainable Source of Energy?. MRS Bull..

[87] Hoehr C., Bénard F., Buckley K., Crawford J., Gottberg A., Hanemaayer V., Kunz P., Ladouceur K., Radchenko V., Ramogida C. (2017). Medical Isotope Production at TRIUMF—From Imaging to Treatment. Phys. Procedia.

[88] Griswold J.R., Medvedev D.G., Engle J.W., Copping R., Fitzsimmons J.M., Radchenko V., Cooley J.C., Fassbender M.E., Denton D.L., Murphy K.E. (2016). Large Scale Accelerator Production of ^225^Ac: Effective Cross Sections for 78-192MeV Protons Incident on ^232^Th Targets. Appl. Radiat. Isot..

[89] Weidner J.W., Mashnik S.G., John K.D., Ballard B., Birnbaum E.R., Bitteker L.J., Couture A., Fassbender M.E., Goff G.S., Gritzo R. (2012). ^225^Ac and ^223^Ra Production via 800 MeV Proton Irradiation of Natural Thorium Targets. Appl. Radiat. Isot..

[90] Weidner J.W., Mashnik S.G., John K.D., Hemez F., Ballard B., Bach H., Birnbaum E.R., Bitteker L.J., Couture A., Dry D. (2012). Proton-Induced Cross Sections Relevant to Production of ^225^Ac and ^223^Ra in Natural Thorium Targets below 200 MeV. Appl. Radiat. Isot..

[91] Cutler C.S. (2020). US DOE Tri-Lab Effort to Produce Ac-225.

[92] Aliev R.A., Ermolaev S.V., Vasiliev A.N., Ostapenko V.S., Lapshina E.V., Zhuikov B.L., Zakharov N.V., Pozdeev V.V., Kokhanyuk V.M., Myasoedov B.F. (2014). Isolation of Medicine-Applicable Actinium-225 from Thorium Targets Irradiated by Medium-Energy Protons. Solvent Extr. Ion Exch..

[93] Mastren T., Radchenko V., Owens A., Copping R., Boll R., Griswold J.R., Mirzadeh S., Wyant L.E., Brugh M., Engle J.W. (2017). Simultaneous Separation of Actinium and Radium Isotopes from a Proton Irradiated Thorium Matrix. Sci. Rep..

[94] Radchenko V., Engle J.W., Wilson J.J., Maassen J.R., Nortier F.M., Taylor W.A., Birnbaum E.R., Hudston L.A., John K.D., Fassbender M.E. (2015). Application of Ion Exchange and Extraction Chromatography to the Separation of Actinium from Proton-Irradiated Thorium Metal for Analytical Purposes. J. Chromatogr. A.

[95] Ramogida C.F., Robertson A.K.H., Jermilova U., Zhang C., Yang H., Kunz P., Lassen J., Bratanovic I., Brown V., Southcott L. (2019). Evaluation of Polydentate Picolinic Acid Chelating Ligands and an α-Melanocyte-Stimulating Hormone Derivative for Targeted Alpha Therapy Using ISOL-Produced ^225^Ac. EJNMMI Radiopharm. Chem..

[96] Robertson A.K.H., McNeil B.L., Yang H., Gendron D., Perron R., Radchenko V., Zeisler S., Causey P., Schaffer P. (2020). ^232^Th-Spallation-Produced ^225^Ac with Reduced ^227^Ac Content. Inorg. Chem..

[97] Augusto R.S., Smith J., Varah S., Paley W., Egoriti L., McEwen S., Goodacre T.D., Mildenberger J., Gottberg A., Trudel A. (2022). Design and Radiological Study of the ^225^Ac Medical Target at the TRIUMF-ARIEL Proton-Target Station. Radiat. Phys. Chem..

[98] Apostolidis C., Molinet R., McGinley J., Abbas K., Möllenbeck J., Morgenstern A. (2005). Cyclotron Production of Ac-225 for Targeted Alpha Therapy. Appl. Radiat. Isot..

[99] Nesteruk K.P., Ramseyer L., Carzaniga T.S., Braccini S. (2019). Measurement of the Beam Energy Distribution of a Medical Cyclotron with a Multi-Leaf Faraday Cup. Instruments.

[100] Higashi T., Nagatsu K., Tsuji A.B., Zhang M.-R. (2022). Research and Development for Cyclotron Production of ^225^Ac from ^226^Ra—The Challenges in a Country Lacking Natural Resources for Medical Applications. Processes.

[101] Morgenstern A., Abbas K., Bruchertseifer F., Apostolidis C. (2008). Production of Alpha Emitters for Targeted Alpha Therapy. Curr. Radiopharm..

[102] Maslov O.D., Sabel’nikov A.V., Dmitriev S.N. (2006). Preparation of ^225^Ac by ^226^Ra(γ, n) Photonuclear Reaction on an Electron Accelerator, MT-25 Microtron. Radiochemistry.

[103] Melville G., Meriarty H., Metcalfe P., Knittel T., Allen B.J. (2007). Production of Ac-225 for Cancer Therapy by Photon-Induced Transmutation of Ra-226. Appl. Radiat. Isot..

[104] Bruchertseifer F., Kellerbauer A., Malmbeck R., Morgenstern A. (2019). Targeted Alpha Therapy with Bismuth-213 and Actinium-225: Meeting Future Demand. J. Labelled Comp. Radiopharm..

[105] IBA and SCK CEN Join Forces to Enable Production of Actinium-225|SCK CEN. https://www.sckcen.be/en/news/iba-and-sck-cen-join-forces-enable-production-actinium-225.

[106] Ermolaev S., Skasyrskaya A., Vasiliev A. (2021). A Radionuclide Generator of High-Purity Bi-213 for Instant Labeling. Pharmaceutics.

[107] Bruchertseifer F., Apostolidis C., Mirzadeh S., Boll R., Murphy K., Morgenstern A. Development of a High-Activity ^225^Ac/^213^Bi Radionuclide Generator for Synthesis of Clinical Doses of ^213^Bi-Labelled Biomolecules. https://publications.jrc.ec.europa.eu/repository/handle/JRC82742.

